# Human cysticercosis in Southeastern Côte d’Ivoire: Seroprevalence and risk factors in two rural departments

**DOI:** 10.1371/journal.pgph.0004634

**Published:** 2025-12-01

**Authors:** Man-Koumba Soumahoro, Kouadio Thierry-Borel N’Dri, Kouassi Eugène Koffi, Mariame Sarranh Fanny, Gildas Boris Tazemda-Kuitsouc, Yves Landry Kangah, Kouadio Narcisse Tano, Kouamé Mathias N’Dri, Cataud Marius Guédé, Offianan André Touré, Stéphane Petres, Allico Joseph Djaman, Mireille Nowakowski, Ronan Jambou

**Affiliations:** 1 Department of Epidemiology and Populations’ Health, Institut Pasteur de Côte d’Ivoire, Abidjan, Côte d’Ivoire; 2 Department of Parasitology Mycology, Institut Pasteur de Côte d’Ivoire, Abidjan, Côte d’Ivoire; 3 Department of Environment and Health, Institut Pasteur de Côte d’Ivoire, Abidjan, Côte d’Ivoire; 4 Recombinant Protein Platform, Institut Pasteur, Paris, France; 5 Unité de Formation et de Recherche des Biosciences, Université Félix Houphouët Boigny, Abidjan, Côte d’Ivoire; Federal University Birnin Kebbi, NIGERIA

## Abstract

Cysticercosis is a parasitic disease caused by *Taenia solium*, primarily transmitted via fecal-oral route. It is a public health concern because it contributes to increasing morbidity of epilepsy. Its true prevalence remains largely unknown in Côte d’Ivoire. This study aimed to estimate the seroprevalence of cysticercosis in rural southeastern Côte d’Ivoire and identify risk factors. Two cross-sectional cluster sample surveys were conducted in villages in the departments of Dabou and Agboville. Data on socio-demographic, neurological history, knowledge about taeniasis/cysticercosis complex and hygiene behaviors’ were collected. A blood sample was collected for serological analysis to detect anti-cysticercosis immunoglobulin G antibodies by ELISA and EITB. The Stata survey command was used for statistical analysis. Qualitative variables were compared using the Chi2 test. A multivariate logistic regression was used to model cysticercosis seropositivity. Cysticercosis seroprevalence was estimated at 9.65% (95%CI: 4.83-18.34%) and 13.11% (95%CI: 9.55-17.74%) in Dabou and Agboville respectively. In Dabou, seropositivity was determined by area of residence and previous knowledge of cysticercosis. The rural population in the Toupah sub-prefecture was more likely to be seropositive (adjusted Odds Ratio [aOR]: 3.53, 95%CI: 1.09-11.44). In Agboville, older adults were 2.74 times more likely to be seropositive than youth at age under 16. Educated individuals (aOR: 0.63, 95%CI: 0.43-0.95), those of muslim faith (aOR: 0.56, 95%CI: 0.34-0.92) and those who reported washing raw vegetables before consumption (aOR: 0.45, 95%CI: 0.22-0.92) had a lower risk. This initial study confirms the circulation of *Taenia solium* and the endemicity of cysticercosis in these rural regions of south-eastern Côte d’Ivoire, area that supply pigs to markets in Abidjan. Insufficient hygiene practices put communities at high risk of fecal-oral transmission. These findings showed public health issue of cysticercosis in rural areas of Côte d’Ivoire, suggesting that targeted interventions should be considered to reduce transmission and associated neurological problems.

## Introduction

Human cysticercosis, a parasitic disease caused by *Taenia solium*, is a significant public health concern in many parts of sub-Saharan Africa. Infection occurs primarily through ingestion of food and water contaminated with parasite eggs [1,2]. Suboptimal hygiene practices facilitate fecal-oral transmission, while traditional pigs farming – marked by close interaction between humans and animals – further promotes the spread of taeniasis and completion the parasite’s lifecycle [3–5]. Once the larva reaches the central nervous system, it can provoke neurocysticercosis with clinical manifestations dominated by epilepsy [5,6]. It is estimated that cysticercosis account for 30% of secondary epilepsy cases in the region [2,7].

Diagnosis of adult tapeworms generally relies on stool analysis. Cysticercosis, particularly in neurological manifestations, is diagnosed using neuroimaging and molecular techniques such as PCR [8]. Where these approaches yield inconclusive results, immunological tests such as Enzyme-linked immunosorbent assay (ELISA) and enzyme-linked immunoelectrotransfert blot (EITB) techniques are essential. The indirect ELISA (Ab-ELISA) detects antibodies indicating past or chronic infection, while the capture ELISA (Ag-ELISA) identifies parasite antigens reflecting active infection. The sensitivity of ELISA ranges from 47.80% to 100.00% and specificity from 81.00% to 96.20%, depending on clinical context (especially the number of cyst) and test methodology [9–11]. This could lead to cross-reaction with other parasites, especially in endemic areas. EITB, by contrast, is more accurate (up to 98.00% sensitivity and 100.00%, specificity), making it suitable for confirming old and chronic infections [12,13].

Since 2010, the World Health Organization (WHO) has recognized cysticercosis as a neglected tropical disease targeted for eradication, reflecting its significant burden: over 18 million people are infected globally and 50,000 deaths annually [14–16]. Underreporting is common in regions with limited diagnostic capacity. Although the disease is rare in Western countries, it remains prevalent in part of Asia, Latin America and Africa, thus posing a substantial public health and economic challenge [14,17–21]. Furthermore, *T. solium* is one of the main causes of death from foodborne diseases, with an estimated burden of 2.80 million DALYs [2].

Numerous risk factors have been identified, including older age [22–24], sex (with variation by setting) [25,26], history of epileptic seizures [22], promiscuity [27], poor sanitation and unsupervised pig farming [23,24,28,29]. The influence of these factors often differs based on to the local context. Human cysticercosis’ seroprevalence also varies widely, depending on the type of immunological tests carried out. The reported estimates include 21.63% in the Democratic Republic of Congo [29], 16.30% in Tanzania [25], 14.30% in Nigeria [22], 12.40% Madagascar [30], 11.90% in Senegal [24], 6.80% in Laos [31] and 5.00% in Vietnam [32].

In Côte d’Ivoire, available data remain scarce and fragmented [33,34]. To date, no studies have measured seroprevalence in rural population, though, high consumption of pork from traditional breeding is suspected to sustain transmission, with lesions were also observed in slaughtered animals carcasses [35,36].

This research is part of a multidisciplinary initiative led by the Institut Pasteur de Côte d’Ivoire following a one health approach [37–39]. The program was designed in response to the concurrent risk of *Schistosoma* and *T. solium* infections in areas where mass treatment campaigns use praziquantel to control schistosomiasis. In the case where neurocysticercosis is present, administering praziquantel can induce severe – sometimes fatal – reactions due to acute brain inflammation. The findings will help identify geographical areas where both parasitic diseases occur simultaneously to adapt prophylaxis campaigns and education strategies.

The primary aim of this study was to estimate the seroprevalence of human cysticercosis in rural communities in two departments of south-eastern Côte d’Ivoire. Additionally, it sought to identify the main risk factors associated with *T. solium* transmission in these populations and to generate epidemiological data necessary for designing public health intervention tailored to the local context.

## Materials and methods

### Study design and setting

Two cross-sectional cluster sample surveys were conducted in villages in the departments of Dabou, between February 10th and April 1st, 2017, and in Agboville, between June 25th and July 11th, 2018.

According to the 2014 census, the population of Dabou and Agboville was estimated to be 148,874 and 292,109 inhabitants respectively [40,41]. Dabou is located between the 5° 19’ north latitude and 4° 23’ west longitude; its vegetation is characterized by dense forest and sub-equatorial climate with annual rainfall of 2000 mm [37]. Agboville is located between latitude the 5° 55’ 41’ North and longitude 4° 13’ 01’‘ West, the climate is tropical, humid with rainfall exceeding 1,500 mm. Its vegetation is varied, ranging from dense plant cover to wooded forest [42].

Populations’ lifestyles in Dabou and Agboville are distinctively incomparable, influenced by their respective environments. Dabou, benefiting from its vicinity to the sea, is characterized by an economic activity based mainly on subsistence agriculture, industrial farming (rubber, oil palm and cocoa), traditional livestock (pigs, goats, sheep), fishing and the traditional processing of cassava tubers into semolina called “Attieke” in local language. In contrast, Agboville, located inland to the north of Dabou, is characterized by a predominantly agricultural economy, with cocoa, coffee, cassava and corn growing predominantly. Traditional pig farming is also practiced by the local populations.

Dabou also encounters specific hygiene and sanitation challenges, related to drinking water access and sanitation. Agboville, on the other hand, benefits from a better-developed hydraulic network, and permits a healthier living environment thanks to its more direct access to drinking water. It should be noted that hygiene and sanitation issues vary according to the geographical and economic specificities of each region.

Both areas are traditional pig farming sites, and main pork meat production sites that supply diverse cities’ animal protein markets of the country.

### Selecting villages and participants

For practical and cost reasons, villages in Agboville and Dabou were selected using cluster sampling based on WHO methodology for vaccine coverage surveys [43]. The sample size for this seroprevalence study was calculated to compare an observed proportion with a reference value from Nigeria (14.30%) [22] considering an alternative proportion of 9.30%. With a two-sided alpha risk of 0.05 and power of 0.90, the minimum sample size needed was 449 participants. The following formula was used to calculate the sample size when comparing an observed proportion to a hypothetical one [44]:


$$n=\;\left[{\left(Z\alpha /2\times \sqrt{}\left(p_{0}\times \left(1-p_{0}\right)\right)+Z\beta \times \sqrt{}\left(p_{1}\times \left(1-p_{1}\right)\right)\right)}^{2}\right]/{\left(p_{0}-p_{1}\right)}^{2}\mathrm{\backslash ]}$$

Where: p_0_: hypothetical proportion (reference prevalence, 0.143); p_1_: alternative proportion (0.093); Zα/2: value from the normal distribution for two-sided alpha risk (for α = 0.05, Zα/2 ≈ 1.96) and Zβ: value from the normal distribution for two-sided alpha risk (for a power of 0.90, Zβ ≈ 1.28)

To address the cluster effect and non-participation, this number was multiplied by 2, with an increase of 15%. As a result, at least 1,033 people were expected to be included per study area.

**Fig 1:**
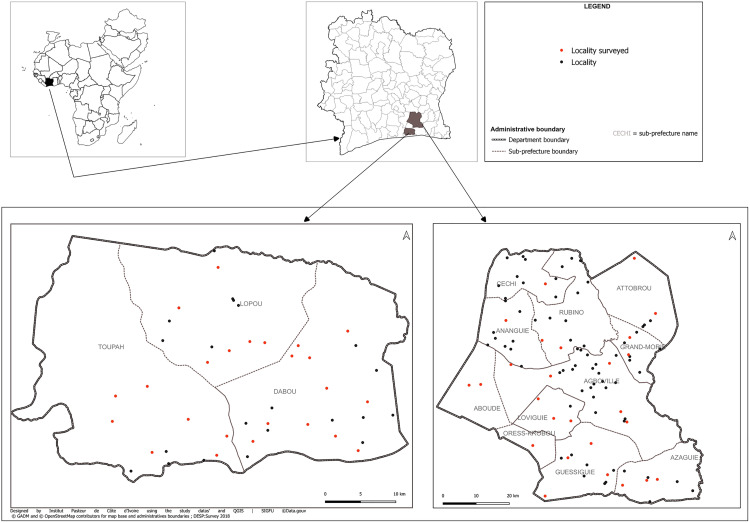
Location of villages selected for the study. The dots represent the villages across the departments, and the red dots represent those selected for the study.

Villages were selected by the cumulative totals’ method according to their populations as counted by health services during the 2015 campaigns of insecticide-treated mosquito net distribution. So, these villages were determined by the residences of the 30 clusters of people randomly selected. Their locations are shown in Fig 1.

Within each village, households were randomly selected using the itinerary method until each cluster had 34 participants. If more than one cluster was selected in a village, the same process was repeated.

Eligibility required participant to be aged five or older, with informed consent, or parentals/guardians informed consent for minors. Children under five years old were not included in the study as venous blood sampling was considered too invasive for them.

### Data collection

A medical team of doctors, nurses and interviewers trained in the study procedures collected the data. The team was assisted by a guide, usually a member of the district health unit -a nurse or community health agent- or a local resident designated by the village.

Data were collected the from selected individuals or their legal representatives who had been previously briefed and who gave their informed consent to participate. Data collection was carried out by a team of medical doctors assisted by an investigator during a face-to-face interview based on a questionnaire provided to the participants. The questionnaire covered socio-demographic characteristics, neurological history, taeniasis/cysticercosis complex knowledge, and hygiene practice. Nurses then collected blood sample using a dry tube. In addition, immediate household environment was also observed. Samples were carried along and pre-stored in a refrigerator at 4°C at reception site before being sent to Institut Pasteur de Cote d’Ivoire. Serum was collected after 3 minutes centrifugation at 3,000 rpm and stored at -20°C in two aliquots until analysis.

### Cysticercosis serology

Detection of antibodies against cysticercosis was performed using Ab-ELISA and Western Blot (EITB) through glycosylated antigens according to the method of Tsang *et al.* [45]. This method is already in use in Côte d’Ivoire for the seroprevalence study of cysticercosis in epileptic patients [39]. Antigens were obtained from infected pig seized in slaughterhouses in Madagascar. Antigen extraction was completed at the Institut Pasteur in Paris using the Tsang *et al.* [45].

The Ab-ELISA technique detects circulating IgG antibodies, indicating prior exposure to the cyst of *T. solium*. It involves reacting antibodies with a cysticerci antigen, followed by revelation with an alkaline phosphatase-labeled antibody. Enzymatic activity produced, proportional to the antibody quantity, was determined by spectrophotometry. The ELISA positivity threshold was calculated per plate. Six negative controls were tested for each plate. Threshold was calculated as the mean optical densities (OD) of the negative controls plus three times the standard deviation of these OD. Samples with an OD equal to the positivity threshold were retested.

The Western Blot (EITB) serology involves detecting *T. solium*-specific glycoproteins on a nitrocellulose paper support, utilizing antibodies present in human serum. This method uses electrophoresis to separate the antigens, transfers them to the support, and then uses an enzymatic immunodetection assay to measure the antibodies specific to cysticercosis. Samples were declared positive if at least 2 of the following 6 bands were enlighten: P6-8, P12, P23-26, P39, P45 and P50-55 [46].

In field conditions, serological diagnosis of cysticercosis using ELISA faces limitations such as cross-reactions with other helminth infections (such as *Echinocccus* or *Schistosoma*) leading to false positives [47]. False negatives can also occur when few cysts are presents or in case of calcified lesions. To improve specificity and reduce risk of false positives, all ELISA-positive samples were systematically confirmed by EITB. This provides more reliable results. So, in this study, samples considered positive tested positive for both ELISA and Western blot.

### Statistical analysis

Double data entry was conducted to minimize the risk of data entry errors and to ensure conformity of the results reported using Epidata 3.1 software (EpiData Association, Odense, Denmark). Data were analyzed using Stata version 16 software (StataCorp LLC, https://www.sata.com). Considering the study design, the Stata’s survey command was used, and a sampling weight was applied to the estimates to reflect the population parameters.

Categorical variables were described by percentages with their 95% confidence intervals (95%CI), and quantitative variables, such as age, by median and interquartile range (IQR). The dependent variable in this study was positive serology for cysticercosis in the participants.

Univariate analysis was performed using the Pearson’s Chi2 design-based to compare categorical variables in seropositive (Cysti+) versus seronegative (Cysti-) participants. Age variable was categorized to determine the age groups most at risk of cysticercosis seropositivity in the study population. There was no particular focus on a specific age group. For the categorical variables selected for the multivariable model, missing data were not imputed but coded as an additional modality labeled “uninformed”. This approach allowed to keep maximum observation number.

A multivariate logistic regression model was built using a stepwise backward procedure to identify the determinants of seropositivity. Explicative variables selected for the model were those associated with the dependent variable (positive serology for cysticercosis) at a p value < 20% in univariate analysis. The final model retained only those variables associated with the dependent variable at the 5% threshold in the bilateral formulation. The final model’s goodness of fit was performed using the Hosmer-Lemeshow test. A p value > 0.05 indicated a model good fit.

### Ethics statement

The *Comité National d’Éthique des Sciences de la Vie et de la Santé* validated the study under approval number 123/MSHP/CNER-dk. This approval was extended under the number 132//MSHP/CNER-km.

The administrative, villages and health authorities were informed in advance of the objectives and modalities of the study through courtesy visits. An information letter and a protocol summary were given to them before the visits, explaining the procedures and announcing the survey date. As a result, the village criers in each village announced the survey date to the population as is common practice, to ensure that it ran smoothly and to raise awareness. Written informed consent was obtained from each participant prior to inclusion in the study. When the participant was under 18, this consent was obtained from parents or guardians.

## Results

### Study population demographics

**Fig 2:**
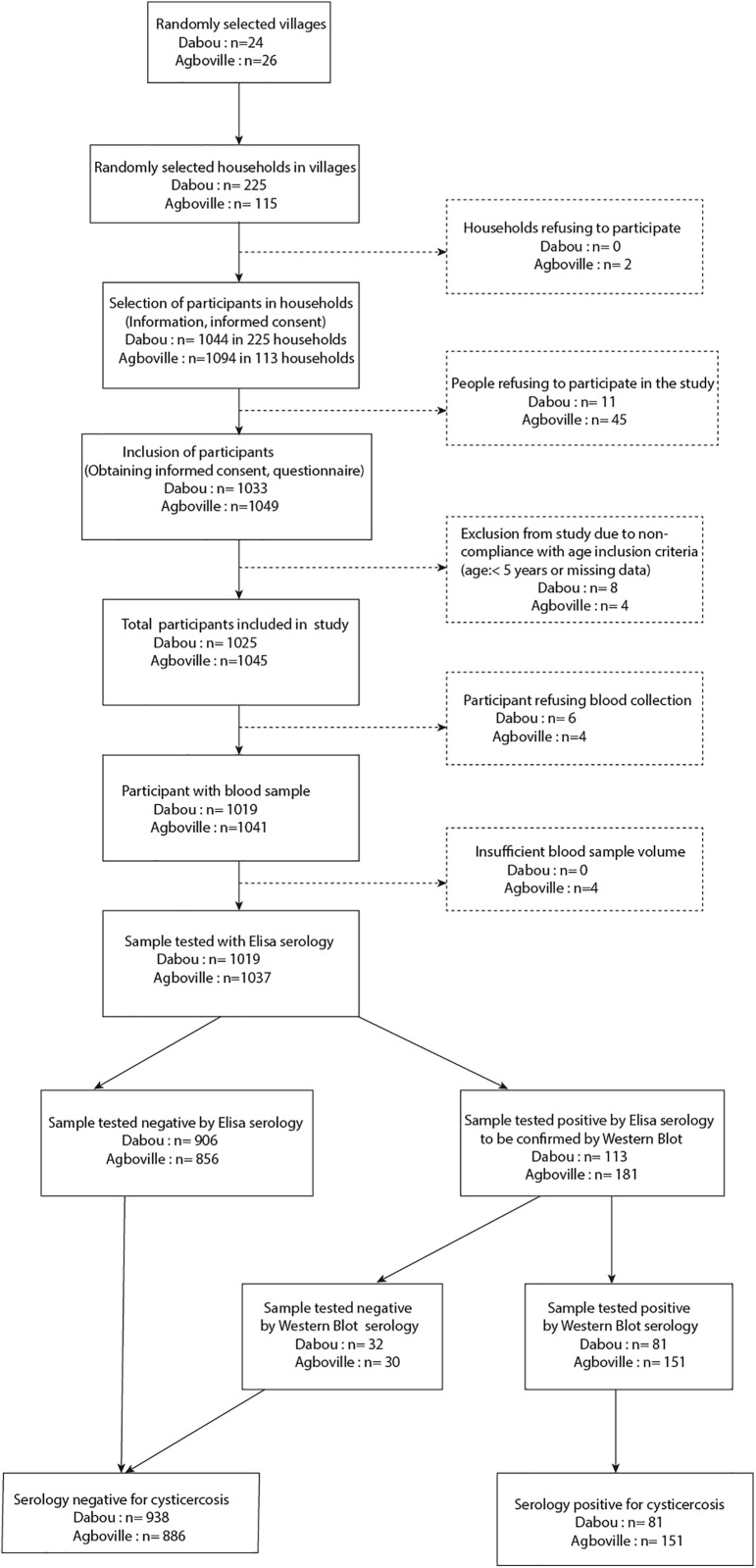
Flow chart of population-based surveys in rural areas in the departments of Dabou and Agboville.

A total of 2,082 people were included in these surveys, with 1,033 persons from 24 villages in Dabou and 1,049 from 26 villages in Agboville. Twelve participants (8 in Dabou and 4 in Agboville) were excluded due to non-compliance with age inclusion criteria (under 5 years old or missing age data). This resulted in a finals samples size of 1025 in Dabou (participation rate: 98.94%) and 1,045 in Agboville participation rate: 95.87%) (Fig 2).

In Dabou, the median age of participants was 33.19 years (IQR 16.10-50.27). Women comprised 53.67% (n = 549) of the participants, 23.51% were under 16 years old and 47.63% had completed elementary education. Participants in Agboville were also predominantly female (sex ratio: 1.19). Over one-third were under 16 years old, and nearly 51% had an elementary education (S1 Table).

Overall, the study population were predominantly female (sex ratio: 1.18) and were younger in Agboville than in Dabou (29.79 vs. 35.32 years; p < 0.001) (S2 Table).

### Seroprevalence of cysticercosis

**Fig 3:**
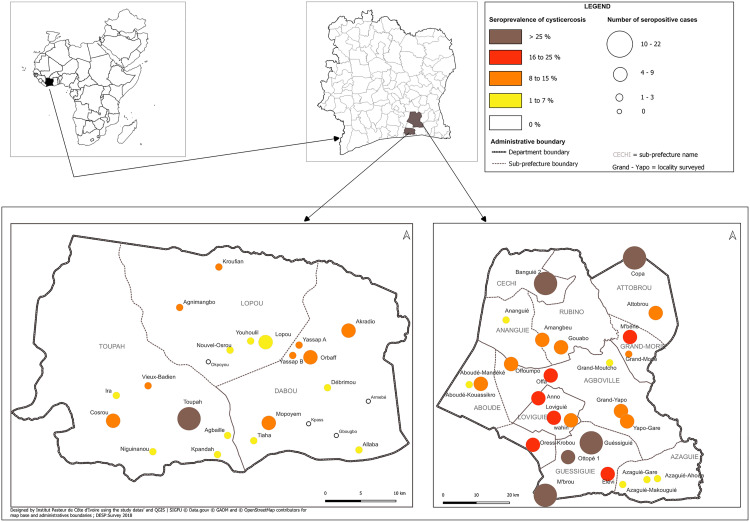
Geographical distribution of cysticercosis seroprevalence in study villages. The circles represent the villages included in the study, which size varies according to the number of cysticercosis seropositive cases. The color of the circle represents the seroprevalence of each village according to the following scale: white circles represent villages with no positive serology; circles are yellow when seroprevalence varies from 1.00 to 7.99%; orange from 8.00 to 15.99%; red from 16.00 to 25.99% and brown when seroprevalence is over 25.99%. The size of the circles is proportional to the number of seropositive cases in the village.

In Dabou, the estimated seroprevalence of cysticercosis was 9.65% (95%CI: 4.83-18.34%) (S1 Table) with village-specific variation ranging from 0 to 33.33%. The highest seroprevalences were recorded in Toupah (33.33%), Mopoyem (14.71%), and Cosrou de (13.29%). In Agboville, the overall seroprevalence was estimated at 13.11% (95%CI: 9.55-17.74%) and varied between 1.47 and 29.73% across villages. The highest frequencies were found in Guessiguié (29.73%), Copa (29.41%) and M’Brou (29.41%) (Fig 3).

In general, seroprevalence was higher in Agboville than in Dabou (13.11% vs. 9.65%; p < 0.001).

### Identifying the determinants of seropositivity

#### Dabou rural population.

Univariate analyses identified several factors associated with seropositivity for cysticercosis: a higher proportion of seropositive individuals were female (65.18% vs. 52.07%, p = 0.031), resided in the Toupah sub-prefecture (62.12% vs. 30.03%, p = 0.004), and had no formal education (35.84% vs. 24.88%, p = 0.042) compared to cysticercosis-negative participants. No significant associations were observed with age (p = 0.170) or Muslim religion (p = 0.134). Neurological history, including notions of recurrent headaches (p = 0.904), epileptic seizures (p = 0.451) and familial epilepsy (p = 0.918) reported by participants, showed no links to seropositivity (S3 Table).

In addition, these univariate analyses revealed no statistically significant association between seropositivity and history of intestinal worms (p = 0.566) or deworming (p = 0.642), knowledge of tapeworms (p = 0.943) and awareness of their mode of transmission (p = 0.814). However, having ever heard of cysticercosis were more frequently reported among seropositive individuals (5.78% vs. 2.27%, p = 0.008) (S4 Table).

Regarding hygiene practices reported by participants, seropositivity was not significantly associated with hand washing (p = 0.517), washing raw vegetables before eating (p = 0.497), drinking tap water (p = 0.144), or eating pork (p = 0.084) (S4 Table). Notably, over half of seropositive individuals reported practicing open defecation (S5 Table).

Multivariate model results demonstrated that residing in the Toupah sub-prefecture was associated with a higher risk of seropositivity (aOR: 3.53, 95%CI:1.09-11.44). Individuals who reported having heard of cysticercosis were three times more likely to be seropositive.

Unexpectedly, those who reported consuming undercooked pork were less likely to be seropositive for cysticercosis compared to those who did not report eating pork (aOR: 0.34, 95%CI:0.16-0.71). This result is surprising given that undercooked pork is traditionally considered a major transmission route for the taeniasis (Table 1).

**Table 1:** Determinants of cysticercosis seropositivity in villages in Dabou department using multivariate logistic regression analysis.

Independent variables	aOR ^(a)^ (95%CI)	p
Sub-prefecture of residence		
Dabou	Reference	
**Toupah**	**3.53 (1.09–11.44)**	**0.037**
Lopou	0.86 (0.40–1.83)	0.679
Have you ever heard of cysticercosis?		
No	Reference	
**Yes**	**3.06 (1.31–7.17)**	**0.012**
Consumption of pork		
No	Reference	
**Yes, undercooked**	**0.34 (0.16–0.71)**	**0.006**
Yes, well cooked	0.81 (0.38–1.70)	0.562

Goodness of fit by Hosmer-Lemeshow test: p = 0.172.

^a^aOR = Adjusted Odd Ratio.

#### Agboville rural population.

In Agboville, univariate analyses did not reveal significant associations between positive serology and sex (p = 0.528), age (p = 0.069), religion (p = 0.081), length of residence (p = 0.698), or area of residence (p = 0.311). The only significant difference was that seronegative individuals were more likely to be educated than seropositive ones (82.89% vs. 74.14%, p = 0.007). There was no significant association with neurological history (recurrent headaches, epileptic seizures or epilepsy in the family). (S6 Table).

Similarly, cysticercosis seropositivity was independent of deworming history (p = 0.943), hand-washing (p = 0.875), washing raw vegetables before consumption (0.078), drinking tap water (0.869), or pork consumption (0.833). Most participants lacked knowledge about tapeworms or had never heard about cysticercosis (S7 Table). Among seropositive participants, a large proportion (72.97%) reported open-air defecation (S5 Table).

Multivariate analysis indicated that people older than 65 (aOR: 2.74, 95%CI:1.47-5.09) and those aged between 26 and 35 (aOR: 1.94, 95%CI: 1.10-3.99) were at a greater risk of seropositivity. Another notable finding in Agboville was that washing raw vegetables before consumption emerged as a protective factor against cysticercosis (aOR: 0.45, 95%CI: 0.21-0.92).

Additionally, education and Muslim religion were identified as a protective factor in Agboville. The protective effect of education (aOR: 0.63, 95%CI: 0.42-0.95) may be linked to improved knowledge and adoption of preventive behaviors. The protective effect of Muslim religion (aOR: 0.56, 95%CI: 0.34-0.92) might be due to religious dietary restrictions regarding pork, which could lower exposure risk (Table 2).

**Table 2:** Factors associated with cysticercosis seropositivity in villages in Agboville department using multivariate logistic regression.

Independent variables	aOR (95% CI)^(a)^	p
Age group		
≤ 15 years	Reference	
16–25 years	0.99 (0.51–1.92)	0.979
**26–35 years**	**1.94 (1.10–3.40)**	**0.023**
36–45 years	1.55 (0.72–3.30)	0.248
46–65 years	1.70 (0.79–3.66)	0.169
** > 65 years**	**2.74 (1.47–5.09)**	**0.003**
Educated		
No	Reference	
**Yes**	**0.63 (0.42–0.95)**	**0.030**
Muslim religion		
No	Reference	
**Yes**	**0.56 (0.34–0.92)**	**0.025**
Washing raw vegetables before consumption		
No	Reference	
**Yes**	**0.45 (0.21–0.92)**	**0.030**
NA	0.75 (0.32–1.75)	0.489

Goodness of fit by Hosmer-Lemeshow test: p = 0.874.

^a^aOR (95% CI) = Adjusted Odd Ratio and 95% confidence interval.

### Environmental factors

Observation of the immediate environment of the households visited indicated that wild garbage dumps were more common in Dabou than in Agboville (38.49% vs. 25.89%; p = 0.020). Conversely, households in Agboville were more likely to have latrines compared to those in Dabou (87.96% vs. 68.90%; p < 0.001) (S8 Table).

Participants who reported respecting hand-washing times were likelier to live in Dabou (35.26% vs. 15.19%; p < 0.001). Most seropositive people who reported open-air defecation lived in Agboville (72.97% vs. 53.75%; p = 0.003). Residents of Dabou were more likely to drink tap water (37.11% vs. 8.52%, p < 0.001), while those in Agboville more often reported rainwater consumption (69.36% vs. 29.31%, p < 0.001). (S5 Table)

## Discussion

This study provided initial data on the local epidemiology of cysticercosis in rural areas in Côte d’Ivoire. Despite its known public health burden, cysticercosis remains understudied in Côte d’Ivoire, particularly in rural areas [2]. Moreover, the WHO has considered cysticercosis an eradicable disease since 2010 [16].

This study revealed the circulation of *T. solium* in rural areas of the Dabou and Agboville departments respectively, confirming the endemicity of cysticercosis in these areas [48]. The observed seroprevalences (Dabou: 9.65%, Agboville: 13.11%) are comparable to other endemic regions such as Madagascar (12.40%) [30] and Peru (13.70%) [49] and Nigeria (14.30%) [22], though they remain lower than seroprevalence reported in Tanzania (16.30%) [25] and the Democratic Republic of Congo (21.63%) [29]. Meanwhile, some areas like Vietnam report lower prevalence (5%) [32], showing that the parasite circulation in these departments is not extremely high or very low when compared to other places in the world. This situation makes clear that the Côte d’Ivoire is facing a public health problem, much like other countries where the disease is common. This initial study confirms the need for larger-scale epidemiological studies in other regions of the country not only to determine the level of *T. solium* circulation but also understand and address the factors driving the parasite transmission.

In both study areas, while no sex-based difference in seropositivity was found, contrasting findings from Tanzania and Guatemala [25,50] suggest that sociocultural roles may mediate gender-related exposure risk, which merits further exploration in the Ivorian context.

In Dabou, age was not a risk factor for seropositivity as demonstrated in a study conducted in the Central Islands of Vietnam [32]. Conversely, in Agboville adults and the elderly showed higher risk, suggesting that cumulative risk of contamination by the parasite and a higher exposure related to daily activities may contribute to seropositivity in population with older age structure. Mwanjali *et al.* have also shown that the seroprevalence of cysticercosis increases with age, getting to the peak in the 36–60 age group [26]. The observed discrepancy between the two study sites shows that health programs should be made to match the age group and daily lives of each area. The findings also indicate that in places with an older people, like Agboville, it is especially important to focus on preventing infection and educating adults and the elderly, as they may be more at risk because of their long-term exposure with the parasite.

In Dabou, the risk of seropositivity was increased with both area of residence and prior knowledge of cysticercosis. During the surveys, some villagers reported pigs roaming around their villages in the past. This observation, coupled with the open-air defecation practice by seropositive individuals, may have been a major factor of amplification of the parasite life cycle [21,51]. This would suggest that strong transmission took place in this area of the department in the past. Open-air defecation, especially by people exposed to *T. solium*, the lack of latrines and the uncontrolled dumping of household waste in the vicinity of households are factors that encourage the perpetuation of fecal-oral transmission. In addition, surveys conducted in the departments of Dabou and Agboville estimated the seroprevalence of porcine cysticercosis at 20.74% and 7.90% respectively; thus confirming that the parasite’s circulation is high in these departments [37]. Local environment observation also revealed that villages in the Toupah sub-prefecture had larger household sizes, than in the other two sub-prefectures of the Dabou department. This could have been increased the likelihood of close contact and facilitate the spread of infection. Consequently, promiscuity could explain why residence is associated with seropositivity in this department, as demonstrated in a study carried out in Madagascar. [30]. Surprisingly, in the department of Dabou, the results showed that the risk was reduced among individuals who reported eating undercooked pork, showing that pork consumption may play a lesser role than environmental exposure. These observations raised the need for targeted intervention focusing on improved sanitation, controlled pig management, and enhanced community education to more effectively interrupt the transmission cycle of T. solium.

In Agboville, the risk was reduced among individuals who were either educated, or of Muslim faith and those who declared that they washed raw vegetables before consumption, as shown in other studies [18]. The link between level of education and seropositivity as demonstrated in a study conducted in Burkina Faso [52], will help determine the type of communication tools to disseminate information during prevention campaigns. People of the Muslim faith generally do not eat pork. Rural populations of these two departments are predominantly non-Muslims, so they can eat pork and may practice traditional pig farming, which could expose them to taeniasis more than Muslims. Furthermore, practice of open defecation among rural populations exposed to *T. solium* is most often found on the banks of watercourses, where wandering pigs go to drink. This increases the risk of exposure for the entire rural population. These findings underscore the importance of culturally adapted health promotion strategies, including targeting non-Muslim pig-raising communities with specific hygiene and sanitation messages to interrupt the parasite life cycle.

Contrary to common assumptions, consumption of pork was not the main driver of infection on these settings. Instead, poor sanitation and fecal-oral transmission appear to play a more critical role, echoing findings from Burkina Faso [52].

This study has some limitations. This may have led to an underestimation of true prevalence due to possible false negatives in ELISA screening step. Additionally, the absence of data on participant migration history limits our ability to determine the precise location acquisition. To ensure internal validity, the study instrument was pre-tested and refined based on feedback from a rural population near Abidjan. In addition, the subjects were randomly included in the study, and the interviews were generally conducted at times when people had finished their activities.

The 2014 General Population Census revealed a male/female sex ratio greater than one, whereas our survey populations were predominantly female. Although the survey sample was predominantly female, likely due to daytime availability, sex was not associated with seropositivity, suggesting minimal bias in this regard.

The strength of this study lies in the fact that, it provides the first data on cysticercosis in Côte d’Ivoire. Indeed, it is the first study that documents the epidemiology of cysticercosis in rural areas of Côte d’Ivoire (Dabou and Agboville); therefore, filling a national data gap on this infection, which is a real public health problem. This study not only documents the epidemiological burden of cysticercosis in rural Côte d’Ivoire but also highlights the urgent need for integrated “One Health” interventions encompassing human health, animal health and environmental sanitation.

## Conclusions

This initial study is one of the very few investigations conducted in Côte d’Ivoire on cysticercosis. The estimated seroprevalence in rural areas reached 9.65% and 13.11% in Dabou and Agboville, both situated in southeastern southern part of the country. These findings confirm the circulation of *T. solium* and the endemicity of cysticercosis in these areas. These endemic rural zones are also major suppliers of pigs to urban markets in Abidjan, raising concerns about broader food safety and disease transmission risks.

The study emphasizes that fecal-oral transmission is the primary route of contamination in rural areas of both departments. It also highlighted the impact of socio-economic factors on the persistence of infection, underscoring the importance of tailored prevention strategies to the context of each community.

To effectively address cysticercosis, an integrated approach is necessary. Key recommendations include increasing public awareness and delivering health education to promote better hygiene practices. Improving infrastructure, particularly on ensuring access to adequate latrines, is identified as a priority. Moreover, implementing strict controls of the pig industry through regular veterinary inspection and the supervision of traditional breeders is considered crucial to minimizing risks.

The study concludes by emphasizing the need for further research involving both humans and pigs. Such investigations should take into consideration social perceptions of hygiene practices to provide a more comprehensive better understanding of the cysticercosis epidemiology and the parasite’s circulation within affected communities.

## Supporting information

S1 Table Socio-demographic characteristics of study participants and seroprevalence of cysticercosis in rural areas in the departments of Dabou and Agboville. (XLSX)

S2 Table Socio-demographic characteristics of study participants by department. (XLSX)

S3 Table Seroprevalence of cysticercosis by socio-demographic characteristics and neurologic history reported by study participants in Dabou. (XLSX)

S4 Table Seroprevalence of cysticercosis according to intestinal worms’ history, personal knowledge of the taeniasis/cysticercosis complex and hygiene habits reported by participants in villages of Dabou’s department. (XLSX)

S5 Table Hygiene habits reported by participants in villages by department. (XLSX)

S6 Table Seroprevalence of cysticercosis by socio-demographic characteristics and neurologic history reported by study participants in Agboville. (XLSX)

S7 Table Seroprevalence of cysticercosis according to intestinal worms’ history, personal knowledge of the taeniasis/cysticercosis complex and hygiene practice conditions reported by participants in villages in the department of Agboville. (XLSX)

S8 Table Households’ immediate environment description by department. (XLSX)
